# A prospective study of plasma levels of asprosin and incident type 2 diabetes in postmenopausal women

**DOI:** 10.1016/j.ajpc.2026.101703

**Published:** 2026-06-13

**Authors:** Bo Yang, Jie Li, Stella Ng, Elizabeth S. Silva, JoAnn E. Manson, Lawrence S. Phillips, Alexander P. Reiner, Atul R. Chopra, Simin Liu

**Affiliations:** a Department of Epidemiology and Biostatistics, Joe C. Wen School of Population and Public Health, Mary and Steve Wen Cardiovascular Division, Department of Medicine, School of Medicine, Center for Global Cardiometabolic Health & Nutrition, University of California, Irvine, CA, USA; b Harrington Discovery Institute, Cleveland, OH, USA; c Brigham and Women’s Hospital, Harvard Medical School, and Harvard T.H. Chan School of Public Health, Boston, MA, United States; d Division of Endocrinology, Emory University, Atlanta, GA, USA; e Departments of Medicine and Surgery, The Warren Alpert Medical School, Brown University, Providence, RI, USA

**Keywords:** Adipokines, Asprosin, Postmenopausal women, Type 2 diabetes, Women's Health Initiative

## 1 Introduction

Identifying women at elevated risk of type 2 diabetes before the onset of overt obesity remains a central challenge in preventive cardiometabolic care [1]. Asprosin, a fasting-induced glucogenic adipokine, stimulates hepatic glucose release and relates to insulin resistance in cross-sectional studies [2,3]. Whether asprosin prospectively predicts incident type 2 diabetes and whether it might serve as an early metabolic signal in women who do not yet appear high-risk by adiposity has not been established. To investigate this, we conducted a large prospective case-control study nested in the ethnically diverse Women’s Health Initiative (WHI).

## 2 Methods

The design of the WHI have been described in detail elsewhere [4]. In brief, between 1993 and 1998, 161,808 postmenopausal women aged 50–79 years were recruited into clinical trials (CT) or observational study (OS). Written informed consent was obtained from all participants, and the study protocol was approved at all participating institutions.

We conducted a prospective nested case-control study within the WHI. Eligible participants were free of diabetes, cardiovascular disease, and cancer (other than non-melanoma skin cancer) at baseline. Using incidence-density sampling, we matched 2602 incident type 2 diabetes cases to 2596 controls in a 1:1 ratio on age, race/ethnicity, clinical center, CT/OS, and time of blood draw. The median follow-up was 13.4 years.

Plasma asprosin was measured using a high-specificity sandwich enzyme-linked immunosorbent assay (ELISA) [2]. Samples from case-control pairs had identical lab procedures and were handled consistently to avoid any systematic difference. The median inter-assay coefficient of variation (CV) was 11.16%.

Baseline characteristics were summarized by case-control status. Multivariable logistic regression estimated odds ratios (ORs) per 1-SD asprosin increase and across quintiles. The adjusted model included age, race/ethnicity, assay batch, CT/OS, physical activity, smoking, alcohol drinking, family history of diabetes, education, menopause age, dietary glycemic load, Alternative Healthy Eating Index (AHEI), total energy intake, region, family income, and BMI. Missing covariates were addressed using multiple imputation. Tests for linear trend across quintiles and restricted cubic spline models for asprosin were performed. The potential interaction effect by BMI status (non-obese, BMI <30 kg/m²; obese, BMI ≥30 kg/m²) was tested with a multiplicative product term.

All analyses were conducted using R. Statistical tests were two-sided, and P values <0.05 were considered statistically significant.

## 3 Results

Cases and controls were similar in age (∼63 years) and race/ethnicity distribution. Women who developed type 2 diabetes had higher BMI, higher dietary glycemic load, lower physical activity, and more frequent family history of diabetes (Fig. 1A).

**Fig. 1:**
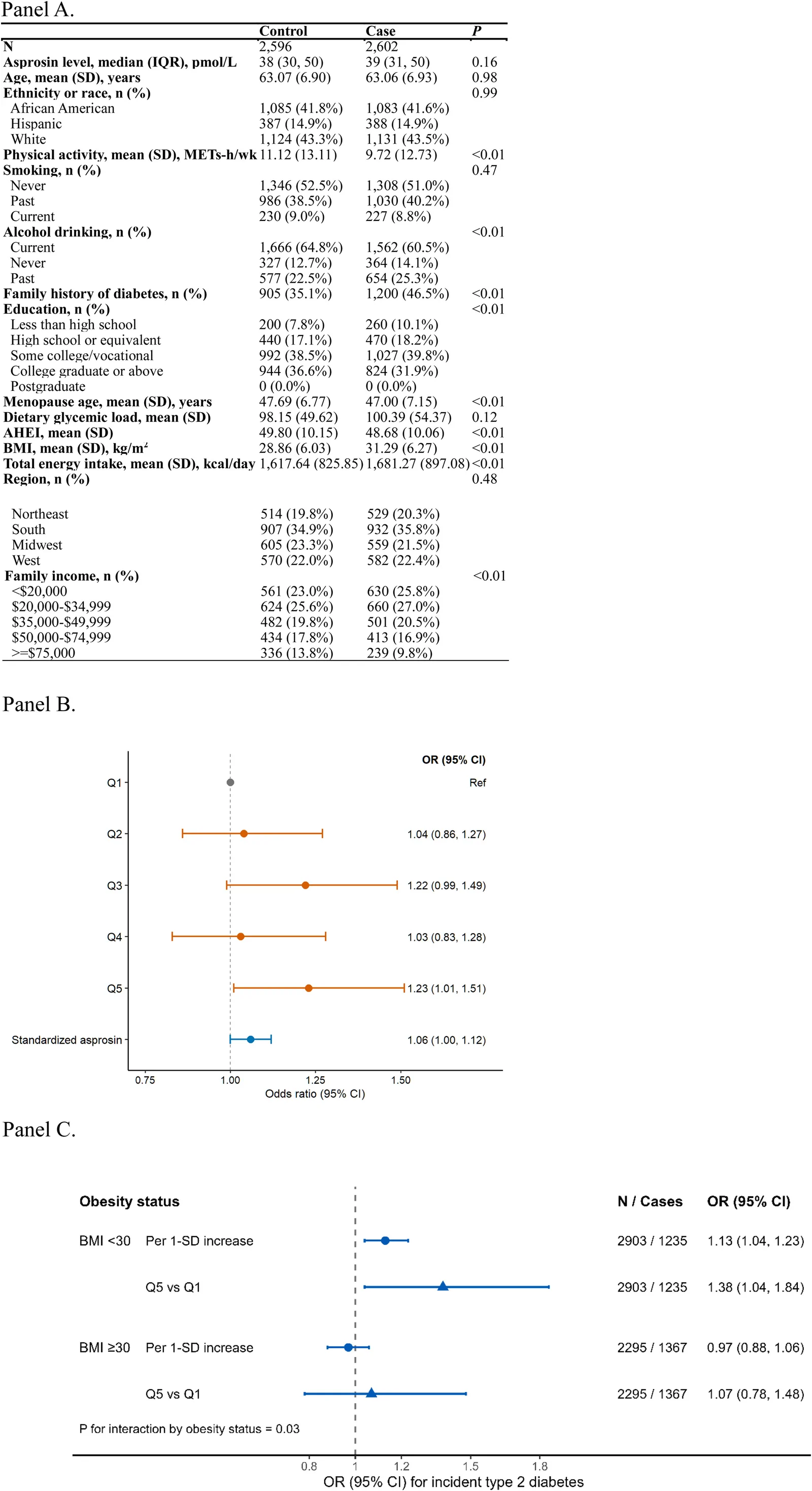
Panel A. Baseline characteristics of cases and controls. Values are presented as mean (SD) for continuous variables, median (interquartile range) for skewed variables, and n (%) for categorical variables. P values were calculated using Welch Two Sample *t*-test for continuous variables, Wilcoxon rank-sum test for skewed variables, and Pearson’s Chi-squared test for categorical variables. Panel B. Associations between Plasma Asprosin Levels and Type 2 Diabetes Risk. Odds ratios (ORs) and 95% confidence intervals (CIs) were estimated using logistic regression models with multiple imputation for missing covariates. Model was adjusted for age, race/ethnicity, batch, trial assignments (CaD, DM, and HT), physical activity, smoking, alcohol intake, family history of diabetes, education, menopause age, dietary glycemic load, Alternate Healthy Eating Index (AHEI), total energy intake, region, family income, and body mass index (BMI). The standardized asprosin estimate represents the OR per 1–SD increase in plasma asprosin levels. Panel C. Interactions by Obesity Status Between Plasma Asprosin and Type 2 Diabetes Risk. Odds ratios (ORs) and 95% confidence intervals (CIs) were estimated using the fully adjusted multivariable logistic regression model. Analyses were stratified by obesity status.

In the fully adjusted model, each 1-SD increase in plasma asprosin was associated with 6% higher odds of type 2 diabetes (OR, 1.06; 95% CI, 1.00–1.12). Women in the highest asprosin quintile had a 23% higher risk than those in the lowest quintile (OR, 1.23; 95% CI, 1.01–1.51; P for trend = 0.05) (Fig. 1B). Restricted cubic splines supported an approximately linear positive association (P for non-linearity = 0.84). Findings were robust after excluding cases diagnosed in the first 3 years, in complete-case analyses, and using log-transformed asprosin.

The association was significantly modified by BMI (P for interaction = 0.03). Among women with BMI <30 kg/m², each 1-SD increase was associated with 13% higher type 2 diabetes risk (OR, 1.13; 95% CI, 1.04–1.23), and women in the highest quintile had 38% higher risk than those in the lowest quintile (OR, 1.38; 95% CI, 1.04–1.84; P for trend = 0.01). In contrast, no association was observed among women with BMI ≥30 kg/m² (Fig. 1C).

## 4 Discussion

In this large, ethnically diverse prospective cohort of postmenopausal women, higher baseline plasma asprosin was independently associated with incident type 2 diabetes, with the association most pronounced among women without obesity.

Asprosin appears to identify a subset of metabolically vulnerable women who would not be flagged as high-risk by adiposity-based screening alone. Among women with BMI <30 kg/m², each 1-SD increment in plasma asprosin was associated with 13% higher odds of T2D, with a clear gradient across quintiles, whereas no association was observed among women with obesity. This pattern is biologically coherent: in women with established obesity, the dominant drivers of diabetes risk likely overshadow the contribution of any single adipokine, while in non-obese women asprosin may reveal early metabolic dysregulation that has not yet manifested as excess weight. This interpretation aligns with our recent companion analysis in the WHI, in which baseline asprosin predicted subsequent 3-year weight gain specifically among postmenopausal women with BMI <30 kg/m² [5]. From a prevention standpoint, this is precisely the population for whom novel biomarkers are most needed, since current clinical risk tools rely heavily on adiposity and frequently miss non-obese women who later develop diabetes. Asprosin may therefore complement existing risk stratification and flag women who appear low-risk by conventional metrics but have underlying glucogenic dysregulation.

Several limitations should be acknowledged. First, plasma asprosin was measured once at baseline. Within-person variability and changes over time were not captured, and any resulting non-differential measurement error would most likely attenuate the observed associations toward the null. Second, as an observational biomarker study, our findings do not establish causality. Asprosin is intertwined with broader metabolic and inflammatory pathways, and residual confounding from unmeasured factors remains possible despite extensive adjustment for covariates. Third, fasting glucose, insulin, HOMA-IR, and HbA1c were not available, which precluded formal evaluation of mediation through insulin resistance or β-cell dysfunction and limited our ability to quantify incremental predictive value beyond standard glycemic biomarkers. Fourth, incident diabetes was based on self-reported diagnosis and medication use. Although this definition has been extensively validated in WHI and any misclassification with respect to baseline asprosin is likely non-differential, some outcome misclassification cannot be excluded. Lastly, the exclusively postmenopausal women cohort is both a limitation for generalizability and a strength. Hormonal transitions and shifts in adipokine physiology around menopause make this population particularly relevant for women's cardiometabolic prevention research, where sex-specific risk markers remain underdeveloped.

In conclusion, higher baseline plasma asprosin levels were prospectively associated with increased type 2 diabetes risk among postmenopausal women, which appeared stronger among women without obesity. Our results identify plasma asprosin as a promising novel biomarker for detecting non-obese postmenopausal women at increased risk of type 2 diabetes, a group that has historically been difficult to identify before clinical disease emerges. Replication in independent cohorts, including men, premenopausal women, and additional racial and ethnic groups, and formal evaluation of how asprosin performs alongside HbA1c, fasting glucose, and established clinical risk scores will be important next steps.

## Funding

The WHI program is funded by the National Heart, Lung, and Blood Institute, National Institutes of Health, U.S. Department of Health and Human Services through 75N92021D00001, 75N92021D00002, 75N92021D00003, 75N92021D00004, 75N92021D00005. Research reported in this work was supported by a grant from the National Institutes of Health (1R01DK125403-01). The study sponsor/funder was not involved in the design of the study; the collection, analysis, and interpretation of data; writing the report; and did not impose any restrictions regarding the publication of the report.

## CRediT authorship contribution statement

**Bo Yang:** Writing – review & editing, Writing – original draft, Visualization, Software, Methodology, Investigation, Formal analysis, Data curation. **Jie Li:** Writing – review & editing, Writing – original draft, Visualization, Software, Investigation, Formal analysis, Data curation. **Stella Ng:** Writing – review & editing, Validation, Software, Investigation, Formal analysis. **Elizabeth S. Silva:** Writing – review & editing, Resources, Data curation. **JoAnn E. Manson:** Writing – review & editing, Validation, Methodology. **Lawrence S. Phillips:** Writing – review & editing, Validation, Methodology. **Alexander P. Reiner:** Writing – review & editing, Validation, Methodology. **Atul R. Chopra:** Writing – review & editing, Resources, Methodology, Data curation. **Simin Liu:** Writing – review & editing, Validation, Supervision, Resources, Project administration, Methodology, Funding acquisition, Data curation, Conceptualization.

## Declaration of competing interest

The authors declare the following financial interests/personal relationships which may be considered as potential competing interests:

Simin Liu reports financial support was provided by National Institutes of Health. If there are other authors, they declare that they have no known competing financial interests or personal relationships that could have appeared to influence the work reported in this paper.
